# rs12329760 Polymorphism in Transmembrane Serine Protease 2 Gene and Risk of Coronavirus Disease 2019 Mortality

**DOI:** 10.1155/2022/7841969

**Published:** 2022-11-22

**Authors:** Saeedeh Sadat Beheshti Shirazi, Fatemeh Sakhaee, Fattah Sotoodehnejadnematalahi, Mohammad Saber Zamani, Iraj Ahmadi, Enayat Anvari, Abolfazl Fateh

**Affiliations:** ^1^Department of Biology, Science and Research Branch, Islamic Azad University, Tehran, Iran; ^2^Department of Mycobacteriology and Pulmonary Research, Pasteur Institute of Iran, Tehran, Iran; ^3^Immunoregulation Research Center, Shahed University, Tehran, Iran; ^4^Department of Physiology, School of Medicine, Ilam University of Medical Science, Ilam, Iran; ^5^Microbiology Research Center (MRC), Pasteur Institute of Iran, Tehran, Iran

## Abstract

The protease produced by the transmembrane serine protease 2 (*TMPRSS2*) gene enhances viral infections and has been linked to severe acute respiratory syndrome coronavirus 2 pathogenesis. Therefore, this study evaluated the association between *TMPRSS2* and coronavirus disease 2019 (COVID-19) mortality. *TMPRSS2* rs12329760 polymorphism was genotyped using the tetraprimer amplification refractory mutation system-polymerase chain reaction method in 592 dead and 693 improved patients. In the current study, the frequency of *TMPRSS2* rs12329760 CC than TT genotypes was significantly lower in improved patients than in dead patients. According to the findings of the multivariate logistic regression test, higher levels of mean age, creatinine, erythrocyte sedimentation rate, C-reactive protein, aspartate aminotransferase, lower levels of 25-hydroxyvitamin D, uric acid, and real-time PCR Ct values and *TMPRSS2* rs12329760 CC genotype were observed to be associated with increased COVID-19 mortality rates. In conclusion, the *TMPRSS2* rs12329760 CC genotype was a polymorphism linked to a significantly higher incidence of severe COVID-19. Further studies are required to corroborate the obtained findings.

## 1. Introduction

A novel coronavirus known as severe acute respiratory syndrome coronavirus 2 (SARS-CoV-2) was identified as the source of a cluster of pneumonia cases in Wuhan, China, at the end of 2019. The virus soon spread throughout the world, triggering a global pandemic of a sickness known as coronavirus disease 2019 (COVID-19) by the World Health Organization (WHO) [1]. Angiotensin-converting enzyme 2 is the host receptor that SARS-CoV-2 binds to (ACE2). In the past, a number of approaches to creating a vaccine against SARS-CoV-2 or MERS-CoV have been tested with varying degrees of success. Since SARS-CoV-2 enters the host cell using the spike (S) protein, it is one of the most popular targets for developing vaccines or treatments for SARS-CoV-2. Efforts are underway to develop a drug to combat the COVID-19 infection. It has been shown in a study that JMX0286, JMX0301, and JMX0941 are the three compounds that are effective allosteric inhibitors of SARS-CoV-2 3CLpro [2, 3].

It has been demonstrated that the effectiveness of three novel oral antivirals (molnupiravir, fluvoxamine, and paxlovid) in lowering the mortality and hospitalization rates in COVID-19 patients. The three oral medications also showed satisfactory overall safety by not increasing the frequency of adverse events. These three oral antiviral medications are still being researched, but the information that is now available indicates that they will offer new hope for COVID-19 recovery and have the potential to be a breakthrough and very promising treatment for COVID-19 [4].

There are several established risk factors related to increased COVID-19 morbidity and mortality, including age, to a lesser extent male gender, and a variety of underlying clinical disorders, such as obesity, diabetes, and cardiovascular disease [5]. The importance of host genetic factors has lately surfaced as a new, although still poorly understood, risk factor for COVID-19. Genome-wide association studies (GWAS) have identified genetic haplotypes spanning in numerous genes that are linked to the severity of COVID-19 [6].

It has been recently demonstrated that SARS-CoV-2 utilizes the angiotensin-converting enzyme 2 (*ACE2*) receptor for host cell attachment, and the viral spike (S) protein is cleaved into S1 and S2 by transmembrane serine protease 2 (TMPRSS2) to allow membrane fusion between the viral and cellular membranes. Because the *ACE2* and *TMPRSS2* are highly expressed in a variety of organs, including the respiratory system, lung, heart, kidney, and gastrointestinal tract, it has been hypothesized that their widespread distribution might increase vulnerability to SARS-CoV-2 infection [7, 8].

There are 496 noncoding genetic and 520 coding genetic variations in the *TMPRSS2* gene region (chr21:42836478-42). Only small percentages of the *TMPRSS2* SNPs were nonsynonymous and had extremely low minor allele frequencies. Of these, 43 SNPs were classified as loss of function (MAF) [9]. There are two nonsilent variations (*TMPRSS2* rs12329760; c.589G > A p.Val160Met and *TMPRSS2* rs75603675; c.23G > T p.Gly8Val) with varying frequencies among the 13 common single-nucleotide polymorphisms (SNPs) within the *TMPRSS2* gene (0.0096 to 0.4 for rs75603675 and 0.172 to 0.385 for rs1232976) [10]. The *TMPRSS2* rs17854725 SNP is a synonymous variant that replaces two isoleucine codons at position 256 [11].

*TMPRSS2* SNPs have been correlated with H1N1 or H1N9 influenza virus infection susceptibility and severity [12]. At the moment, SNPs that affect gene expression or protein function are often discussed in the context of COVID-19 in literature reviews and in silico investigations based on genotyping data from existing databases [13, 14].

Due to its functional significance, the missense variant rs12329760, which changes the amino acid valine at position 160 to methionine, might be of importance in the context of COVID-19 [15]. The rs12329760 T-allele is linked to the numerous copies of the TMPRSS2-ERG gene fusion, which is linked to poorer survival and greater tumor recurrence in prostate cancer [16].

Based on the aforementioned connections, this study examined the effect of the *TMPRSS2* variation rs12329760 on the probability of SARS-CoV-2 infection and the COVID-19 mortality rate in Iranian patients.

## 2. Materials and Methods

### 2.1. Study Participants

The present study was conducted with the approval of the Ethics Committee of Pasteur Institute of Iran (IR PII REC 1400.042). The study participants provided written informed consent.

Enrollment started on May 11, 2020, and ended on January 28, 2022. The patients were considered SARS-CoV-2 positive if at least one real-time reverse transcription polymerase chain reaction (real-time RT-PCR) result was positive. In total, 1,285 COVID-19-positive patients were included in this study according to exclusion criteria, such as human immunodeficiency virus (HIV), cancer, chronic kidney disease, heart disease, pregnancy, diabetes, liver disease, cystic fibrosis, asthma, chronic obstructive pulmonary disease, and obesity. All samples of patients were collected from one hospital in a city with the same ethnic group that was the inclusion criteria.

All clinical parameters of the patients were examined as soon as they were referred to the hospital. The clinical parameters, including real-time PCR cycle threshold (Ct) values, 25-hydroxyvitamin D, erythrocyte sedimentation rate (ESR), C-reactive protein (CRP), platelets, white blood cells (WBC), triiodothyronine (T3), thyroxine (T4), thyroid-stimulating hormone (TSH), alkaline phosphatase (ALP), aspartate aminotransferase (AST), alanine aminotransferase (ALT), high-density lipoprotein (HDL), low-density lipoprotein (LDL), triglyceride (TG), cholesterol, fasting blood glucose (FBS), uric acid, and serum creatinine, were extracted from the patients' medical records.

### 2.2. DNA Extraction and *TMPRSS2* rs12329760 Genotyping

Genomic DNAs were extracted from peripheral blood mononuclear cells (PBMCs) using High Pure PCR Template Preparation Kit (Roche Diagnostics Deutschland GmbH, Mannheim, Germany), according to the manufacturer's instructions.

We designed the tetraprimer amplification refractory mutation system-polymerase chain (T-ARMS–PCR) method for genotyping of the *TMPRSS2* rs12329760 polymorphism.

The primers of *TMPRSS2* rs12329760 were designed using the PRIMER1 website (http://primer1.soton.ac.uk/primer1.html). The primer sequences were then evaluated for heterodimers, self-dimers, GC content, and hairpins using the oligo-analyzer. For facilitating reliable discrimination between two alleles, the mismatched nucleotide was added at the third position from the 3′-end terminal of the primers (Table 1).

The PCR was carried out in a 20 *μ*L reaction volume containing 10 *μ*L TEMPase Hot Start DNA Polymerase (Ampliqon, Hamburg, Germany), 50 ng of genomic DNA, 1.0 *μ*L of 10 pmol of outer forward primer, 2.5 *μ*L of 10 pmol of outer reverse primer, 2.5 *μ*L of 10 pmol of inner forward primer, and 1.0 *μ*L of 10 pmol of inner reverse primer. Amplification was performed using the PCR conditions 95°C for 20 minutes, followed by 38 cycles of 95°C for 35 seconds, annealing at 62°C for 30 seconds and 72°C for 35 seconds, followed by final extension at 72°C for 10 minutes. The PCR amplicons were analyzed on 1.5% agarose gel (Supplementary Figure 1).

For the assessment of the efficiency and accuracy of the assay, DNA sequencing by ABI 3500 DX Genetic Analyzer (ABI, Thermo Fisher Scientific, Waltham, MA, USA) was performed on a subset of PCR-amplified samples, and the results of T-ARMS- PCR were compared to those obtained by sequencing (Supplementary Figure 2).

### 2.3. Statistical Analyses

The chi-square (*Χ*^2^) test was utilized to assess the frequency of *TMPRSS2* rs12329760 genotypes in dead and improved patients. Additionally, the Mann-Whitney *U* test was employed to evaluate the data for continuous variables. Multiple logistic regression models were employed to determine the association of genotypes, allele frequencies, and other variables with the possibility and mortality of COVID-19. The odds ratio (OR) and the accompanying 95% confidence interval (CI) were calculated. The effect of *TMPRSS2* rs12329760 on COVID-19 mortality was determined using the area under the receiver operating characteristic curve (AUC-ROC) analysis. All the tests were two sided, and a *P* value < 0.05 was considered statistically significant. The calculations were performed using IBM SPSS version 22.0. (SPSS. Inc., Chicago, IL, USA). The inheritance mode analysis in SNPStats software was applied to assess the association between COVID-19 mortality and *TMPRSS2* rs12329760. The Akaike information criterion (AIC) and Bayesian information criterion (BIC) were used to choose the model with the greatest match for each SNP. The *Χ*^2^ test was employed to assess the Hardy-Weinberg equilibrium (HWE) for genotype distributions [17].

## 3. Results

### 3.1. Demographic Characteristics of COVID-19 Patients

Table 2 shows the demographic information of COVID-19 patients. The current study included 1,285 patients with COVID-19, 592 and 693 of whom were in the dead and improved groups, respectively. The mean (±SD) age values of dead and improved patients were 58.0 ± 11.4 and 51.7 ± 12.8 years, respectively. In this study, 677 (52.7%) and 608 (47.3%) were male and female, respectively. The mortality of the disease increased with mean (±SD) age (*P* < 0.001); high levels of Cr (*P* < 0.001), FBS (*P* < 0.001), ALP (*P* < 0.001), ALT (*P* < 0.001), AST (*P* < 0.001), ESR (*P* < 0.001), and CRP (*P* < 0.001); and lower levels of cholesterol (*P* < 0.001), TG (*P* = 0.001), LDL (*P* < 0.001), HDL (*P* < 0.001), 25-hydroxyvitamin D (*P* < 0.001), real-time PCR Ct value (*P* = 0.011), and uric acid (*P* < 0.001).

### 3.2. Correlation between *TMPRSS2* rs12329760 and COVID-19 Mortality

**Figure 1:**
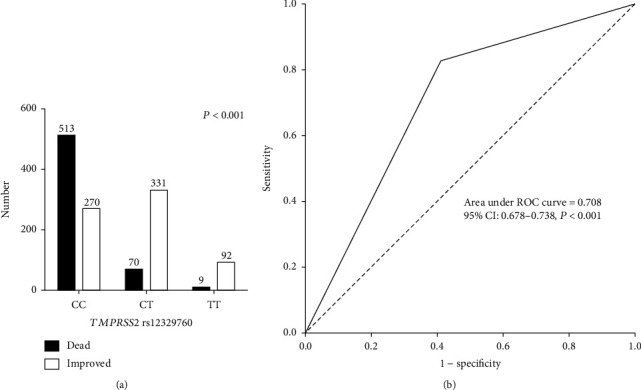
(a) Frequency of *TMPRSS2* rs12329760 in COVID-19 patients. (b) ROC curve with the *TMPRSS2* rs12329760 for prediction the mortality rate in COVID-19 patients.

Figure 1 shows the effect of *TMPRSS2* rs12329760 on COVID-19 infection mortality. The patients with *TMPRSS2* rs12329760 CC genotypes demonstrated significantly higher COVID-19 infection mortality than individuals with other genotypes; nevertheless, COVID-19-recovered patients had TT genotypes (Figure 1(a)).

The *TMPRSS2* rs12329760 inheritance model, including codominant, dominant, recessive, and overdominant, was generated using SNPStats software. For *TMPRSS2* rs12329760, the best-fitting inheritance model was codominant with the lowest AIC and BIC values. A higher risk of death was associated with the CC genotype.

*TMPRSS2* rs12329760 genotypes were compatible with HWE in improved (*P* = 0.570) and dead (*P* = 0.281) patients. MAF (T-allele) in improved, dead, and all patients was 0.37, 0.07, and 0.23, respectively (Table 3).

Furthermore, the AUC-ROC values for *TMPRSS2* rs12329760 were 0.708, indicating that host genetic variables frequently contribute to viral infection mortality (Figure 1(b)).

### 3.3. Risk Factors Linked to COVID-19 Mortality

This study examined several parameters linked with COVID-19 infection mortality using multivariate logistic regression analysis. The mortality of COVID-19 infection was correlated with mean age (OR 0.975, 95% CI 0.958-0.993, *P* = 0.006), HDL (OR 1.046, 95% CI 1.025-1.066, *P* < 0.001), LDL (OR 1.024, 95% CI 1.019-1.030, *P* < 0.001), uric acid (OR 2.043, 95% CI 1.768-2.361, *P* < 0.001), creatinine (OR 0.074, 95% CI 0.038-0.145, *P* < 0.001), ESR (OR 0.965, 95% CI 0.951-0.979, *P* < 0.001), CRP (OR 0.973, 95% CI 0.963-0.984, *P* = 0.004), 25-hydroxyvitamin D (OR 1.057, 95% CI 1.038-1.077, *P* < 0.001), AST (OR 0.983, 95% CI 0.973-0.992, *P* < 0.001), real-time PCR Ct values (OR 0.961, 95% CI 0.932-0.991, *P* = 0.011), and *TMPRSS2* rs12329760 CC (OR 16.723, 95% CI 10.841-25.798, *P* < 0.001) (Table 4).

## 4. Discussion

This comprehensive study was performed in Iran to evaluate a possible correlation between *TMPRSS2* rs12329760 polymorphism with the susceptibility of SARS-CoV-2 acquisition and disease progression.

To date, most in silico reports have conducted to investigate the impact of polymorphisms in the *TMPRSS2* gene on COVID-19 [13, 18]. In a pilot study conducted on a small scale, the *TMPRSS2* rs12329760 T-allele carriers' frequency was significantly lower in Italian SARS-CoV-2-positive patients than in a European reference group from the Gnom Aggregation Database (GnomAD) [19]. Interestingly, a significant higher prevalence of T-allele carriers was observed in dead patients (0.07) than in improved patients (0.37). The allele frequency of *TMPRSS2* rs12329760 in this study is 0.23. This value is equal to the one reported in GnomAD for East Asia (0.38), Finns (0.37), Estonia (0.31), Sweden (0.29), and Africans (0.29) [20]. This would be reasonable if it is assumed that T-allele carriers express more genes that could result in increased *TMPRSS2* activity leading to increased infection susceptibility [15].

The *TMPRSS2* rs12329760 polymorphism was categorized as deleterious based on the data collected from computational research utilizing Sorting Intolerant From Tolerant (SIFT), screening for nonacceptable polymorphisms (SNAP2), PolyPhen-2, and PROVEAN [21]. This was due to the fact that splice variations resulting from genetic variations in TMPRSS2 have modified the expression of *TMPRSS2*, altering susceptibility to infection with SARS-CoV-2 [22]. According to bioinformatics studies (ConSurf), the mutation (rs12329760) might impair the TMPRSS2 protein's stability. Protein stability has an effect on the functional and structural activities of a protein. Any alteration in protein stability results in misfolding or abnormal protein structure [23].

Furthermore, because extremely damaging nonsynonymous SNPs have high conservation scores, evolutionary conservation analysis in the protein sequence is required to evaluate whether a mutation is null or has any effect on the protein [24]. The results of ConSurf revealed that the variant is located in conserved regions; therefore, it might increase the probability of COVID-19 infection by downregulating *TMPRSS2* [25]. The p.V160M mutation may alter the function of *TMPRSS2* gene. A role in ligand and/or protein interaction has been hypothesized for the scavenger receptor cysteine rich (SRCS), which is a highly conserved domain whose function is not entirely understood. It is interesting to note that various proteins involved in host defense, including cluster of differentiation 5 (CD5), CD6, and complement factor I, contain this domain, therefore increasing the probability that the *TMPRSS2* rs12329760 role in COVID-19 infection may extend beyond its peptidase activity associated with viral protein priming [26, 27]. Molecular dynamics modeling confirms structural distortions caused by a full shift in the motif, resulting in changes in the protein's quaternary structure and a concurrent rise in B factor with the V160M variation. Although the V160M is not positioned in the catalytic pocket of *TMPRSS2*, a decrease in protein stability may hinder SARS-CoV-2 viral entry; nevertheless, this needs to be validated [27].

In this study, higher risk of death was associated with the *TMPRSS2* rs12329760 CC genotype than with other ones. In line with the aforementioned finding, a meta-analysis study has shown that COVID-19 patient with *TMPRSS2* rs12329760 CC genotype compared to individuals with CT and TT genotypes had a 1.79-fold greater chance of getting infectious symptoms (moderate to severe) [28]. The detailed analysis of the COVID-19 Host Genetic Initiative (HGI) data revealed an important link between the *TMPRSS2* rs12329760 polymorphism and disease severity [29, 30]. The first whole-genome investigation in 322 Chinese patients infected with COVID-19 revealed a decreased allele frequency of the *TMPRSS2* rs12329760 variant in patients with severe disease than patients with moderate disease, demonstrating the critical role of *TMPRSS2* in progression of COVID-19 [31]. According to a previous study, this polymorphism might have contributed to the spread of COVID-19 among the Italian population, which had a high mortality rate and poor prognosis [18]. Moreover, it was demonstrated that *TMPRSS2* rs12329760 had a predictive value for identifying individuals at risk of developing severe COVID-19 [20]. In contrast, Wulandari et al. discovered no correlation between the *TMPRSS2* rs12329760 polymorphism and the severity of COVID-19 infection but found a significant correlation between the *TMPRSS2* rs12329760 CC genotype and COVID-19 mortality [32].

The mortality of COVID-19 infection was correlated with AST, lipid profile, uric acid, creatinine, 25-hydroxyvitamin D, and real-time PCR Ct values. Patients with COVID-19 frequently had abnormal liver functions. Epidemiological research revealed that nearly half of patients suffered from varying degrees of liver impairment [33, 34]. Numerous reports discovered that liver enzyme levels were significantly higher in intensive care unit (ICU) patients than in non-ICU patients [35, 36]. Additionally, it was demonstrated that moderate and severe COVID-19 individuals with abnormal liver function were more likely to have a liver injury. However, abnormal liver functions might be related to the severity of COVID-19 infection in patients. This could be due to the scattering of genes *ACE2* and *TMPRSS2* in various tissues such as the liver [37].

Three indicators of renal damage, creatinine, urea nitrogen, and uric acid are observed to be positively linked with the probability of death in COVID-19 patients. Several cells in the kidney can coexpress both *ACE2* and *TMPRSS2* genes. The data of the single-cell RNA-seq from Gene Expression Omnibus dataset (GSE134355) indicated that both *ACE2* and *TMPRSS2* expression levels were high in epithelial cells, endothelial cells, mesangial cells, kidney podocytes, and nephron epithelial cells of the kidney suggesting that SARS-CoV-2 might directly attach to ACE2-positive cells in the kidney, impairing renal tubule function [37, 38].

The 25-hydroxyvitamin D might downregulate the mRNA expression of the *TMPRSS2* gene significantly in the treated mice. The aforementioned findings imply that 25-hydroxyvitamin D has a molecular effect on SARS-CoV-2 entrance genes, preventing or reducing SARS-CoV-2 virus transmission within lung epithelial cells. As a result, the 25-hydroxyvitamin D deficiency is correlated with the severity of COVID-19 infection [39].

In this study, the dead patients who had *TMPRSS2* rs12329760 CC genotype indicated lower level of real-time PCR Ct values with high viral load. Wulandari et al. observed that patients carrying the *TMPRSS2* rs12329760 CC genotype, which indicates the presence of valine amino acid, typically have a lower Ct value [32]. There is a possibility that this genotype is associated with a higher viral load and high mortality rate.

The limitations of this study were lack of examination of other effective polymorphisms in *TMPRSS2* gene, and the effects of this SNP on *TMPRSS2* expression were not studied.

In conclusion, the C allele of the common *TMPRSS2* rs12329760 polymorphism confers an increased risk of COVID-19 mortality rate. Furthermore, clinical parameters, including AST, LDL, HDL, uric acid, 25-hydroxyvitamin D, and real-time PCR Ct values, were linked to the severity of infection. However, the findings of the present study might develop new ideas about how to use *TMPRSS2* rs12329760 SNP as a predictor or biomarker of COVID-19 severity or clinical results.

## Figures and Tables

**Table 1:** Sequences and melting temperatures of the *TMPRSS2* rs12329760 primers.

SNP	Primer	Primer sequence	Size (bp)	T_m_	Annealing temp
*TMPRSS2* rs12329760 (C/T)	OF	5′-GGACAAATTCCACCTGCTGGTTATAGG-3′	—		62°C
	OR	5′-GTGCCTTGAGAAGAGTGAACTGTGC-3′	701	66°C	
	IF	5′-GCCAGGACTTCCTCTGAGATGAGTAAA**T**-3′	487	66°C	
	IR	5′-GGGACCAAACTTCATCCTTCCG**G**-3′	275	66°C	

The specific nucleotides at 3′-end of primers are in bold, while the mismatches are underlined. SNP: single-nucleotide polymorphism; OF: outer forward primer; OR: outer reverse primer; IF: inner forward primer; IR: inner reverse primer.

**Table 2:** Comparison laboratory parameters between dead and improved patients infected with COVID-19.

Variables	Dead patients (*n* = 592)	Improved patients (*n* = 693)	*P* value
Mean age ± SD	58.0 ± 11.4	51.7 ± 12.8	<0.001^∗^
Gender (male/female)	315/277 (53.2/46.8%)	362/331 (52.2/47.8%)	0.728
ALT (IU/L) (mean ± SD) (reference range: 5-40)	44.4 ± 23.9	33.1 ± 24.1	<0.001^∗^
AST (IU/L) (mean ± SD) (reference range: 5-40)	37.0 ± 13.1	31.2 ± 15.6	<0.001^∗^
ALP (IU/L) (mean ± SD) (reference range: Up to 306)	202.1 ± 66.1	169.3 ± 90.1	<0.001^∗^
Cholesterol (mg/dL) (mean ± SD) (reference range: 50-200)	116.7 ± 39.7	122.2 ± 36.3	<0.001^∗^
TG (mg/dL) (mean ± SD) (reference range: 60-165)	116.2 ± 41.5	132.0 ± 41.7	0.001^∗^
LDL (mg/dL) (mean ± SD) (reference range: up to 150)	69.8 ± 32.2	107.6 ± 49.3	<0.001^∗^
HDL (mg/dL) (mean ± SD) (reference range: >40)	30.7 ± 10.9	34.3 ± 11.6	<0.001^∗^
WBC (10^9^/L) (mean ± SD) (reference range: 4000-10000)	7544.1 ± 2655.9	7718.8 ± 2894.1	0.506
CRP (mg/L) (mean ± SD) (reference range: <10 mg/L negative)	67.5 ± 21.4	56.8 ± 20.8	<0.001^∗^
ESR (mm/1st h) (mean ± SD) (reference range: 0-15)	55.5 ± 15.5	46.5 ± 15.4	<0.001^∗^
FBS (mg/dL) (mean ± SD) (reference range: 70-100)	111.0 ± 43.4	105.6 ± 41.3	<0.001^∗^
Platelets × 1000/cumm (mean ± SD) (reference range: 140000-400000)	185 ± 77	183 ± 66	0.587
Uric acid (mg/dL) (mean ± SD) (reference range: 3.6-6.8)	3.7 ± 1.2	5.7 ± 1.6	<0.001^∗^
Creatinine (mg/dL) (mean ± SD) (reference range: 0.6-1.4)	1.1 ± 0.3	0.8 ± 0.3	<0.001^∗^
T3 (ng/dL) (mean ± SD) (reference range: 2.3-4.2)	3.1 ± 1.2	2.5 ± 0.8	0.062
T4 (mcg/dL) (mean ± SD) (reference range: 5.6-13.7)	8.9 ± 4.1	8.3 ± 3.8	0.222
TSH (mu/L) (mean ± SD) (reference range: 0.4-4.5)	3.4 ± 1.7	3.2 ± 1.5	0.557
25-hydroxy vitamin D (ng/mL) (mean ± SD) (sufficiency: 21-150)	26.9 ± 10.2	35.9 ± 13.6	<0.001^∗^
Real-time PCR Ct values	12.8 ± 7.0	26.6 ± 8.1	0.011^∗^

ALT: alanine aminotransferase; AST: aspartate aminotransferase; ALP: alkaline phosphatase; TG: triglyceride; LDL: low-density lipoprotein; HDL: high-density lipoprotein; WBC: white blood cells; CRP: C-reactive protein; ESR: erythrocyte sedimentation rate; FBS: fasting blood glucose; T3: triiodothyronine; T4: thyroxine; TSH: thyroid-stimulating hormone; Ct: cycle threshold; SD: standard deviation. ^∗^Statistically significant (<0.05).

**Table 3:** *TMPRSS2* rs12329760 association with COVID-19 mortality.

		Groups					
Model	Genotype	Improved patients (*n* = 693)	Dead patients (*n* = 592)	OR (95% CI)	*P* value	AIC	BIC
Allele	C	871 (63.0%)	1096 (93.0%)	—	—	—	—
	T	515 (37.0%)	88 (0.7%)	—	—	—	—

Codominant	C/C	270 (39.0%)	513 (86.7%)	1.00	<0.0001	1448.8	1469.5
	C/T	331 (47.8%)	70 (11.8%)	0.11 (0.08-0.15)			
	T/T	92 (13.3%)	9 (1.5%)	0.05 (0.03-0.10)			

Dominant	C/C	270 (39.0%)	513 (86.7%)	1.00	<0.0001	1451.8	1467.3
	C/T-T/T	423 (61.0%)	79 (13.3%)	0.10 (0.07-0.13)			

Recessive	C/C-C/T	601 (86.7%)	583 (98.5%)	1.00	<0.0001	1707.3	1722.8
	T/T	92 (13.3%)	9 (1.5%)	0.10 (0.05-0.20)			

Overdominant	C/C-T/T	362 (52.2%)	552 (88.2%)	1.00	<0.0001	1573.5	1598.0
	C/T	331 (47.8%)	70 (11.8%)	0.15 (0.11-0.20)			

Minor allele frequency (T)		0.37	0.07	—	—	—	—

OR: odds ratios; CI: confidence intervals; AIC: Akaike information criterion; BIC: Bayesian information criterion.

**Table 4:** Factors associated with dead patients infected with COVID-19.

Factors		
Baseline predictors	OR (95% CI)	*P* value
Mean age ± SD	0.975 (0.958-0.993)	0.006^∗^
HDL (mg/dL)	1.046 (1.025-1.066)	<0.001^∗^
LDL (mg/dL)	1.024 (1.019-1.030)	<0.001^∗^
Uric acid (mg/dL)	2.043 (1.768-2.361)	<0.001^∗^
Creatinine (mg/dL)	0.074 (0.038-0.145)	<0.001^∗^
ESR (mm/1st h)	0.965 (0.951-0.979)	<0.001^∗^
CRP (mg/L)	0.973 (0.963-0.984)	0.004^∗^
25-hydroxyvitamin D (ng/ml)	1.057 (1.038-1.077)	<0.001^∗^
AST (IU/L)	0.983 (0.973-0.992)	<0.001^∗^
Real-time PCR Ct values	0.961 (0.932-0.991)	0.011^∗^
*TMPRSS2* rs12329760 (CC)	16.723 (10.841-25.798)	<0.001^∗^

HDL: high-density lipoprotein; LDL: low-density lipoprotein; ESR: erythrocyte sedimentation rate; CRP: C-reactive protein; AST: aspartate aminotransferase; Ct: cycle threshold; *TMPRSS2*: transmembrane protease 2; SD: standard deviation; OR: odds ratios; CI: confidence intervals; ^∗^Statistically significant (<0.05).

## Data Availability

The data used to support the findings of this study are included within the article and supplementary file.

## Supplementary Materials

Supplementary Materials Supplementary Figure 1: the result of *TMPRSS2* rs12329760 genotyping with T-ARMS-PCR Ladder 100 bp. Lane No. 1 is genotype TT (701 bp and 487 bp), Lane No. 5 is genotype CT (701 bp, 487 bp, and 275 bp), and Lane No. 8 is genotype CC (701 bp and 275 bp). Supplementary Figure 2: the sequencing results of *TMPRSS2* rs12329760 genotypes for confirming the T-ARMS-PCR method (10% of samples randomly were sequenced). (Supplementary Materials)
