# Supplementary material for: rs12329760 Polymorphism in Transmembrane Serine Protease 2 Gene and Risk of Coronavirus Disease 2019 Mortality
Source: Biomed Res Int. 2022 Nov 22;2022:7841969. doi: 10.1155/2022/7841969 (PMC9708353; doi:10.1155/2022/7841969)
Supplement: Supplementary Materials — Supplementary Figure 1: the result of TMPRSS2 rs12329760 genotyping with T-ARMS-PCR Ladder 100 bp. Lane No. 1 is genotype TT (701 bp and 487 bp), Lane No. 5 is genotype CT (701 bp, 487 bp, and 275 bp), and Lane No. 8 is genotype CC (701 bp and 275 bp). Supplementary Figure 2: the sequencing results of TMPRSS2 rs12329760 genotypes for confirming the T-ARMS-PCR method (10% of samples randomly were sequenced). (Supplementary Materials) [file 7841969.f1.docx]

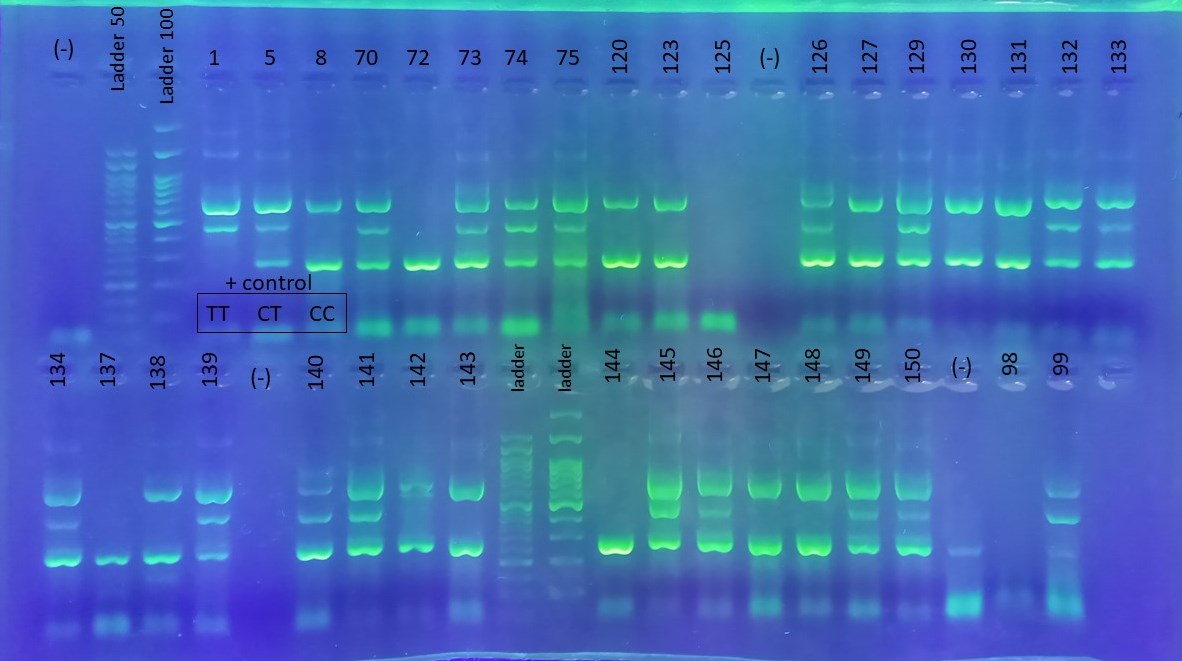


**Supplementary Figure 1:** The result of *TMPRSS2* rs12329760 genotyping with T-ARMS-PCR. Ladder 100 bp. Lane No. 1, is genotype TT (701 bp and 487 bp), Lane No 5 is genotype CT (701 bp, 487 bp, and 275 bp), and Lane No. 8 is genotype CC (701 bp and 275 bp).


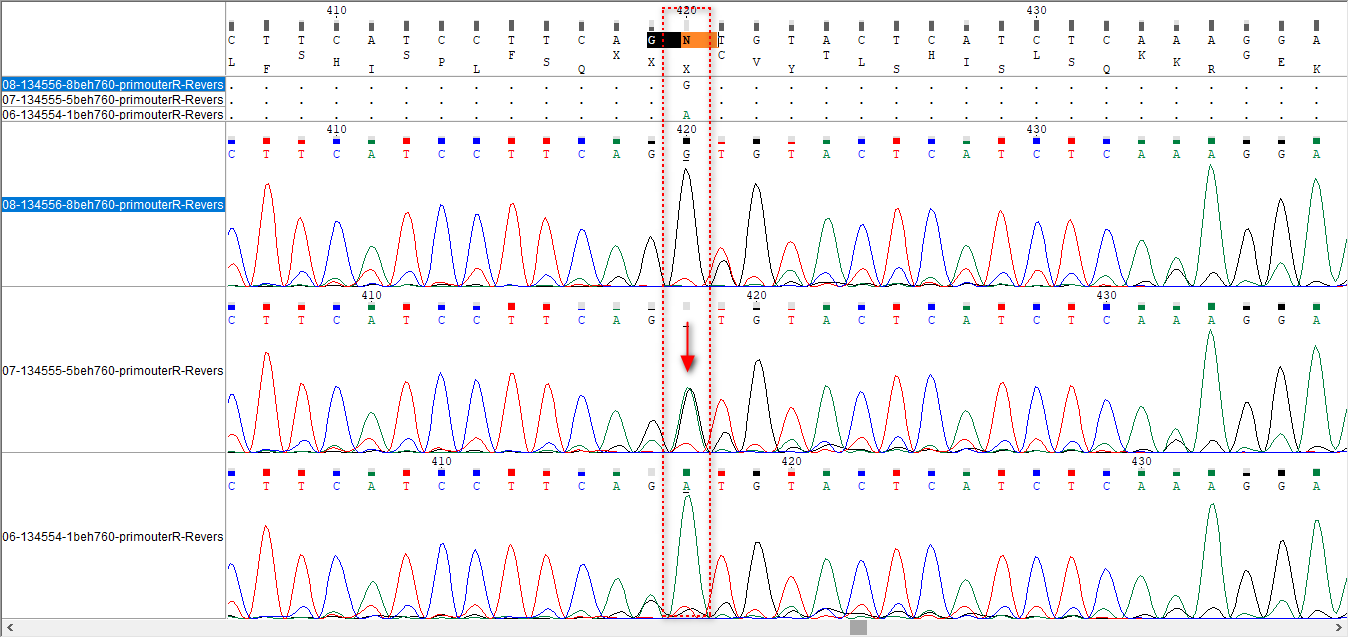


**Supplementary Figure 2:** The sequencing results of *TMPRSS2* rs12329760 genotypes for confirming the ARMS-PCR method (10% of samples randomly were sequenced)
